# To the Future and Beyond: Recent Advances in the Application of Percutaneous Coronary Intervention

**DOI:** 10.3390/jcm10020177

**Published:** 2021-01-06

**Authors:** Shun Kohsaka

**Affiliations:** Department of Cardiology, Keio University School of Medicine, Shinanomachi 35, Shinjuku‐ku, Tokyo 160‐8582, Japan; sk@keio.jp

We are very fortunate to be practicing interventional cardiology during an era of rapid clinical and technological evolution, which allows us to offer potentially life-saving options for challenging cardiac conditions. The development of coronary stents, imaging techniques to better visualize lesions and repairs, and new adjunctive antithrombotic agents and regimens are very active areas of research. In addition, the development of risk scores and models has led to improved accuracy in the prediction of procedure-related mortality, and complications such as bleeding or acute kidney injury. Despite these advances, the burden of coronary artery disease, both in the chronic and acute phase, remains high. In this Special Issue, we collected original and review articles that: (1) assessed short- and long-term clinical outcomes as a consequence of various advances in this area, (2) highlighted unmet needs, and (3) discussed future research directions.

## 1. Advances in PCI for Stable Ischemic Heart Disease

The classic risk prediction model used for stable ischemic heart disease (SIHD) has been repeatedly debated, and the value of percutaneous coronary intervention (PCI) is often questioned in low- to moderate-risk patients. More recently, the International Study of Comparative Health Effectiveness with Medical and Invasive Approaches (ISCHEMIA) trial failed to show a reduction in hard clinical endpoints with an early invasive strategy in SIHD with moderate or severe ischemia [1]. Niimi et al. tested the applicability and potential influence of this trial in daily practice [2]. They extracted data of 13,223 consecutive SIHD patients who underwent PCI during the ISCHEMIA study period and demonstrated that 77.3% of SIHD patients with moderate to severe ischemia were eligible for the trial. However, caution is needed in the extrapolation of the study results for non-eligible patients (e.g., patients with left main (LM) lesions), since the authors found that patients not eligible for the ISCHEMIA trial had high-risk baseline characteristics and demonstrated poorer 2-year outcomes than eligible patients. 

Patients with a complex coronary anatomy are prone to be excluded from enrollment in clinical trials such as the ISCHEMIA, and require individual assessment. Nakachi et al. aimed to quantify the “survival benefit” in patients undergoing PCI for chronic obstructive thrombosis (CTO) [3]. Among the 2625 patients who underwent CTO-PCI at 65 centers, effect modifiers for the association between successful procedure and one-year mortality included diabetes, multivessel disease, Canadian Cardiovascular Society class ≥ 2, and prior myocardial infarction (MI). When each of these modifiers was assigned a single point in the integer scoring system, the patients with scores ≥ 3 showed a profound difference in one-year mortality between subgroups of patients with successful and failed PCI treatment (11.3%), whereas this difference was minimal in patients with a score < 2 (0.7%). Furthermore, Winter et al. demonstrated that in CTO-PCI, the presence of LM lesions is associated with excess mortality [4], and specifically, the CTO of the circumflex artery was particularly vulnerable. These studies underscore the need for the personalized assessment of CTO patients with particular focus on long-term outcomes. Another issue of debate regarding patients with impaired left ventricular ejection fraction (LVEF) is the possible benefits of cell-based therapy in treating patients who are non-candidates for PCI. In a pilot-phase randomized clinical trial of 60 patients, autologous CD34+ cell therapy gradually and effectively improved LV systolic function in patients with diffuse CAD and preserved LVEF [5]. Although hypothetical at this point, cell-based therapy can become an effective and safe alternative therapy for patients with severe diffuse CAD in the near future.

Recently, there has been increasing interest in the early revascularization of subgroups of patients with heart failure. In the small, high-risk subgroup in the ISCHEMIA trial with heart failure and LVEF of 35% to 45%, an initial invasive approach was associated with better event-free survival [6]. However, upon analysis of a pooled database of five nationwide prospective registries from 55 centers in Korea, Kang et al. demonstrated that complete revascularization reduced three-year adverse events only in patients with preserved LVEF. These findings require confirmation with larger datasets or future prospective studies. 

## 2. Advances in PCI for Acute Coronary Syndrome

In the management of acute coronary syndrome (ACS), particularly ST-elevation myocardial infarction (STEMI), the time to coronary flow restoration is vital for survival and overall outcomes. However, there exists significant variation between the reported cases and the application of PCI by operators, institutions, and regions. Randomized controlled studies have shown a trend towards greater clinical benefit with PCI when implemented as an early invasive strategy in higher risk patients, and Ikemura et al. demonstrated that the broader use of an early invasive strategy comes at the cost of an increased risk of acute kidney injury, suggesting that the pre-procedural risk–benefit profile of ACS should be assessed thoroughly [7]. ACS is known to have a broad clinical spectrum with large variations in the incidence of in-hospital mortality [8]. There is a marked discrepancy between the patients’ predicted risk and the use of PCI in real-world practice [9], and the risk of unintended adverse outcomes associated with PCI should not be underestimated.

Furthermore, the procedural complication rate is high in these patients, and the implementation of strategies to avoid complications (e.g., bleeding) remains suboptimal. As for the use of antithrombotics, recent RCTs have indicated that aspirin, widely recognized as a key antiplatelet agent in this area, has limited additional clinical benefits when associated with P2Y12 inhibitors. Guedeney et al. evaluated the safety and efficacy of early aspirin discontinuation with P2Y12 inhibitor single antiplatelet therapy continuation, as compared to a strategy of sustained dual antiplatelet therapy (DAPT) following ACS or PCI [10]. Their meta-analysis, comprising a total of 40,621 patients from very recent randomized trials, demonstrated that the early discontinuation of aspirin, with or without concomitant OAC treatment, was associated with a significant reduction in bleeding events without detectable impacts on mortality or ischemic risk.

The transradial approach is another key strategy for the avoidance of bleeding; however, radial artery spasm (RAS) remains one of the most common complications, and may lead to patient discomfort and procedural failure during these procedures. Birgy et al. investigated the impact of inhalation sedation with a 50% nitrous oxide/oxygen premix [11]. Although this inhalation sedation was proven to be safe, no significant differences were observed in the occurrence of RAS and pain scale scores when compared to traditional opioid analgesic administration. Notably, the female sex, a BMI < 25 kg/m^2^, puncture difficulty, the use of a plastic needle, and a 6F sheath were identified as independent predictors of RAS. On a separate note, vasospasm in the coronary artery was found to be useful for predicting the prognosis after Type II MI events [12].

## 3. Volume-Outcome Relationships

The public reporting of PCI has recently undergone significant refinement, considering the large body of evidence suggesting that the mandated reporting of outcomes is associated with the aversion of high-risk patients, and may worsen overall outcomes. However, the impact of PCI volume in relation to absolute surgical volume has not been studied extensively. In a nationwide registry-based analysis involving 220,934 patients, surgical CABG volume was associated with patients’ clinical outcomes after PCI when a limited number of PCIs were performed at the facility (e.g., less than 200/year) [13]. The findings suggest that seeking an ideal balance between surgical and interventional revascularization procedures may result in improved outcomes. We must also reconsider how to compromise between identifying metrics that are simple and convenient, and those that more clearly capture overall procedural quality. 

## 4. Final Words

The final manuscript was a topic that brought us back to the fundamentals of PCI: an approach towards balloon preparation. One of the major complications of PCI procedures is incomplete stent apposition, and air in the balloon might lead to this technical complication. Kreuser et al. tested 114 balloons four times using three different methods, and found that the best method to connect a PCI balloon to the inflation syringe while removing air was by using a three-way valve.

The editor hopes that this issue will be of interest to not just interventional cardiologists who perform PCI, but also to a broader range of clinicians that encounter coronary artery disease.
